# Asymptomatic Bacterial Vaginosis in Pregnancy and Missed Opportunities for Treatment: A Cross-Sectional Observational Study

**DOI:** 10.1155/2019/7808179

**Published:** 2019-05-02

**Authors:** Nkosinathi Joyisa, Dhayendre Moodley, Thandeka Nkosi, Raesetja Talakgale, Motshedisi Sebitloane, Megeshnee Naidoo, Quarraisha Abdool Karim

**Affiliations:** ^1^Department of Obstetrics and Gynaecology, School of Clinical Medicine, University of KwaZulu-Natal, Durban, South Africa; ^2^Centre for the AIDS Programme of Research in South Africa, Durban, South Africa

## Abstract

**Background:**

High rates of bacterial vaginosis (BV) have been described in nonpregnant South African women. Studies of BV in South African pregnant women are sparse. Diagnosis and prompt treatment of BV in pregnancy are expected to have a positive impact on pregnancy outcomes and HIV prevention. This study was undertaken to determine the prevalence of BV in pregnant women in a high HIV burden periurban setting in KwaZulu-Natal and explore how to enhance BV diagnosis in this setting where syndromic management of sexually transmitted diseases is the standard of care.

**Methods:**

In this cross-sectional study, consenting HIV uninfected pregnant women were examined for abnormal vaginal discharge; nurses determined the vaginal pH and collected a vaginal swab for Gram-stain and Nugent scoring.

**Findings:**

Among 750 HIV uninfected pregnant women, 280 (37.3%; 95%CI 33.9-40.9) tested positive for BV. Using a vaginal pH > 4.4, 65% of women with BV were correctly identified, while an abnormal vaginal discharge correctly identified a significantly lower proportion (52.9%) of women with BV (p=0.005). The sensitivity, specificity, and positive and negative predictive values of vaginal pH testing were 65.9% (95%CI 60.0 – 71.5%), 61.4% (95%CI 56.8 – 65.9%), and 50.1% and 75.4%, respectively. The 20-24 year-old pregnant women were twice more likely to test positive for BV than the adolescent pregnant women (43.6% vs 21.1%) (p = 0.037) and BV was not associated with the duration of a sexual relationship, frequency of unprotected sex during pregnancy, number of lifetime sex partners, or the partner's age.

**Conclusion:**

There is a high burden of primarily asymptomatic BV in HIV uninfected pregnant women in this periurban setting. Both the sensitivity and specificity of vaginal pH testing are superior to the symptomatic diagnosis of BV but not good enough to be used as a screening tool.

## 1. Introduction

Bacterial vaginosis (BV), characterized by a deficiency of beneficial hydrogen peroxide producing* Lactobacillus *spp. in the vaginal microbiome, increased alkalinity, and spurious foul-smelling vaginal discharge, is common in women of reproductive age worldwide [1]. The prevalence of BV in sub-Saharan Africa, a region with the highest antenatal HIV prevalence and high incidence of adverse pregnancy outcomes, ranges from 20 to 50% in women of reproductive age [2].

BV in pregnancy is clinically significant because of its association with pre-term labor and delivery, pre-mature, pre-labor rupture of membranes, spontaneous miscarriage, and infections post-delivery [3–6]. BV may also pose a risk for the acquisition of sexually transmitted infections (STIs) such as HIV,* N. gonorrhoeae*,* C. trachomatis*, and HSV-2 that are independently associated with preterm delivery if untreated [7–12].

Clinically BV has been diagnosed based on a fishy odor of vaginal discharge, or the presence of clue cells on microscopy or a vaginal pH ≥ 4.5 [13]. The gold standard for BV diagnosis is a laboratory based method using Nugent score criteria that quantifies the* Lactobacillus *sp. relative to other bacterial morphotypes [14]. Treatment of BV is based on the presence of abnormal vaginal discharge and the standard syndromic management of sexually transmitted infections (STIs) include a 2g stat dose of Metronidazole for BV and vaginal candidiasis or 500mg twice daily for seven days in the event symptoms persist [15]. Using this treatment approach, and as with other STIs in pregnancy, asymptomatic pregnant women mostly go untreated [16]. Furthermore, it has been shown that the long course antibiotic treatment is a better treatment option to achieve better cure rates [17].

Diagnosis and prompt treatment of BV in pregnancy is expected to have a positive impact on preventing acquisition of HIV and other STIs during pregnancy. Diagnosis of BV would also be useful in identifying pregnant women at substantial risk for HIV infection, who in turn may be offered Pre-Exposure Prophylaxis (PrEP) and enhanced risk reduction counselling.

This study was undertaken to determine the prevalence of BV in HIV uninfected pregnant women attending primary health clinics in a high HIV burden periurban setting in KwaZulu-Natal and explore how to enhance BV diagnosis in this setting where syndromic management of sexually transmitted diseases is the standard of care.

## 2. Methods

This cross-sectional study was part of a larger HIV incidence cohort study that enrolled HIV uninfected pregnant women at three primary health care clinics in a high antenatal HIV prevalence setting in South Africa between February 2017 and March 2018. Pregnant women who tested HIV negative at their first antenatal visit at three primary health clinics in Umlazi, a periurban township in KwaZulu-Natal with an antenatal HIV prevalence of 36%, were screened for enrolment into the parent study. Consenting HIV uninfected women meeting the parent study eligibility criteria that included confirmed pregnancy, <28 weeks' gestation, and intent to reside in the catchment area for the next 12 months were enrolled.

Demographic and sexual-behavioral questionnaires were administered by a trained interviewer. A research nurse performed all study related clinical investigations. Participants were screened for sexually transmitted infections as per the National Guidelines for STI Management and treated accordingly [15]. During genital examination for vaginal discharge, the research nurse collected three vaginal swabs and determined vaginal pH using a pH-Fix 3.6-6.1 PT test strip (Macherey-Nagel, GmbH & Co, Germany). The vaginal swabs were transported to a commercial laboratory (Global Clinical Virology Laboratory, Durban, South Africa) for further processing and storage. The first swab was used to make a slide smear for Gram staining and Nugent Scoring for the diagnosis of BV. The remaining two swabs were stored for further investigations. Slides were read by a single experienced technician. The laboratory recently scored 100% on the CAP EQA for diagnosis of bacterial vaginosis.

A Nugent score was based on the ratio of large Gram positive rods (*Lactobacillus* spp.) relative to small Gram negative variable or curved rods (*Gardnerella* spp./*Mobiluncus* spp.). A predominance of large Gram positive rods (*Lactobacillus* spp.) was suggestive of a normal vaginal microbiome. A Nugent score of 0-3 was reported as Normal, 4-6 as Intermediate, and 7-10 as BV. We further subcategorized BV into moderate (score 7-8) and severe (score 9-10) [14].

Data were captured in real-time in the parent study database (iDatafax Version 2014.1.1) and included demographic characteristics, gravidity, gestational age, clinical symptoms of STIs, vaginal pH, sexual behavioral data, socioeconomic data, and laboratory results including the BV Nugent Score and BV microscopy outcomes.

The data were analyzed using Stata 13.0 (StataCorp (2013). Stata Statistical Software: Release 13. College Station, TX: StataCorp LP). The proportion of women who tested positive for BV and the associated 95% confidence intervals were calculated. Significant associations between categorical outcomes and categorical explanatory variables were assessed using a chi-square (*χ*^2^) test. We used the Kruskal Wallis test to compare means of dependent variables. Sensitivity and specificity analysis of the pH was evaluated to identify the cutoff value for optimal diagnostics. All statistical tests were conducted at 5% level of significance.

Institutional regulatory oversight was provided by the University of KwaZulu-Natal Biomedical Research Ethics Committee (BE 194/18).

## 3. Results

A total of 750 women with a mean age of 23.6 years (range 15-46 years) were enrolled. The majority (>80%) were neither married nor living with their sexual partners; 24% of the HIV uninfected pregnant women were adolescents (95%CI 21.1-27.2), 61% of whom were in a sexual relationship with men in the 20-24-year age group and 22% had much older sexual partners (>25 years). Consistent and correct condom use during pregnancy was reported by < 1% of the pregnant women and the median number of unprotected sexual acts during the 1st or 2nd trimester of pregnancy was 6 (IQR 3-12).

Using the Nugent score criteria, 280 (37.3%; 95%CI 33.9-40.9) women tested positive for bacterial vaginosis (Nugent score 7-10) among whom 47.1% were asymptomatic and 121 (16.1%; 95%CI 13.7-18.9) had an intermediate score (Nugent score 4-6). Among the women with BV, 90 (32%) cases were further classified as severe BV (Nugent score 9-10) and 43% of these women were asymptomatic (Table 1).

Using a vaginal pH > 4.4 as a diagnostic marker, 65% of women with BV were correctly identified, while an abnormal vaginal discharge correctly identified a significantly lower proportion (52.9%) of women with BV (p=0.005). Overall, the proportion of women with a vaginal pH > 4.4 increased with an increase in the Nugent score (53.4% for a score of 4-6, 64% for a score of 7-8, and 70% for a score of 9-10) (Table 1). The mean vaginal pH in pregnant women with a normal Nugent score (score 0-3) was 4.4±0.5 and 33.2% (113/340) of the women had an uncharacteristically high vaginal pH between 4.7 and 6.1. The sensitivity and specificity of vaginal pH testing in this pregnant population were 65.9% (95%CI 60.0 – 71.5%) and 61.4% (95%CI 56.8 – 65.9%), respectively. The positive and negative predictive values for a vaginal pH > 4.4 as a screening tool were 50.1% and 75.4%, respectively.

In this periurban cohort of HIV uninfected pregnant women, the prevalence of BV was significantly higher in older women than adolescent pregnant women (p=0.037). In particular, the 20-24-year pregnant women were twice more likely to test positive for BV than the adolescent pregnant women (43.6% vs 21.1%). BV was not associated with the duration of a sexual relationship, frequency of unprotected sex during pregnancy, number of lifetime sex partners, or the partner's age (Table 2). The prevalence of BV in adolescent (≤19 yrs) pregnant women with a sexual partner of the same age group was 34%, 30.3% if they had a sexual partner in the 20-24-year old age group, and 38.5% if their sexual partners were older than 25 years. There was no significant trend in the BV prevalence in association with disparate age groups (p=0.751).

**Figure 1:**
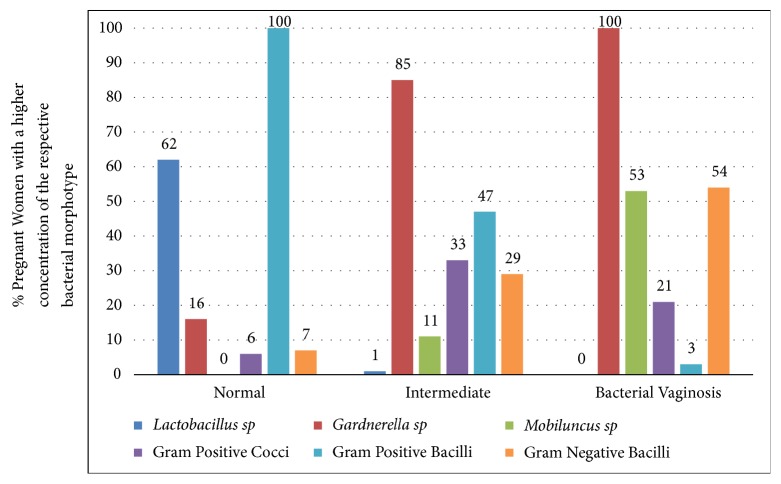
Bacterial profile in pregnant women with normal Nugent score (0-3) (n=349), intermediate score (4-6) (n=121), and bacterial vaginosis (Nugent score 7-10) (n=280).

Using Gram stained microscopy all (100%) pregnant women with a normal vaginal microbiome tested positive for Gram positive bacilli as opposed to 47% of women with an intermediate score and 3% of women with BV (Figure 1). By enumerating the number of bacterial morphotypes per oil field, a* Lactobacillus* spp. dominance was demonstrated in 62% of women with a normal score, 1% of women with an intermediate score, and none in the women with BV. While* Gardnerella *spp. are quite prominent in women with an intermediate score (85%) or BV (100%),* Mobiluncus *spp. are found to be in abundance in 53% of women with BV and not found in women with a normal vaginal microbiome.

## 4. Discussion

Using Nugent scoring on vaginal swabs collected by trained research nurses, a third of HIV uninfected pregnant women attending primary health clinics in a periurban setting in South Africa tested positive for BV. Approximately 47% of the positive BV cases were asymptomatic. The overall performance of vaginal pH testing in identifying 66% of women with BV was superior to using abnormal vaginal discharge alone as an indication of BV, the latter correlated with 53% of the women with BV. Bacterial characterization by morphotypes which indicated* Lactobacillus* spp. depletion in 38% of women with a normal Nugent score has provided a plausible explanation for the uncharacteristically high vaginal pH in these women. Both the sensitivity and specificity of vaginal pH testing are superior to the symptomatic diagnosis of BV but not good enough to be used as a screening tool. Perhaps a combination of vaginal pH testing with a higher sensitivity and a Whiff test with a higher specificity is needed to be evaluated against the syndromic management of BV and STIs in high BV prevalence settings like South Africa. Madhivanan et al. demonstrated that a combination of vaginal pH testing and the Whiff test correctly identified 82% of laboratory diagnosed BV cases in a young sexually active nonpregnant Indian population with a high BV prevalence (45.1%; 95%CI 41.5-52.8) [18]. Dadhwal et al. also suggested a combination of POC tests viz vaginal pH and the Whiff test in asymptomatic women to improve specificity of diagnosing BV in pregnancy [19].

The high prevalence of BV (37.3%; 95%CI 33.9-40.9) in this HIV uninfected pregnant population in South Africa is consistent with other sub-Saharan African pregnant populations (Zimbabwe 32.6%, Zambia 48.3%) [20, 21] and other South African studies of nonpregnant women at risk for HIV acquisition [22–24]. Our study is the first large cohort study of 750 HIV uninfected pregnant women of all ages attending primary health care clinics in a high HIV burden subdistrict of KwaZulu-Natal. A recent study of 220 South African antenatal attendees reported a much lower BV prevalence (17.7%) [25], and possible reasons for the variation in BV prevalence could be the smaller sample size, women were recruited from an antenatal clinic in a tertiary hospital, and vaginal swabs were self-collected by participants.

Risk factors for BV in nonpregnant women include having multiple sex partners, having a new sex partner, and a lack of condom use as evident by the highest prevalence of BV among female sex workers and HIV infected women [26, 27]. Importantly, these studies further underscored the strong association between BV and recent unprotected sexual intercourse as indicated by the presence of prostate specific antigen in the vaginal fluid. In our cohort of HIV uninfected pregnant women, none of the behavioural characteristics iterated in the above studies were associated with BV. The high BV prevalence in the 19- to 24-year-old pregnant women is consistent with a South African rural community survey of nonpregnant women but the BV prevalence in the younger nonpregnant women was higher (41.1%) than that reported for our periurban pregnant population in the same age group (32.8%) [24].

Abnormal vaginal discharge is associated with BV,* Candida *spp., and* Trichomonas vaginalis*; however consistent with other studies in pregnant and nonpregnant women, nearly 50% of pregnant women with BV in our study were asymptomatic [26–28]. Using a vaginal discharge screening algorithm chart as part of the syndromic approach to managing reproductive tract infections in pregnant women, Tann et al. (2006) also concluded that 50% of BV infections are missed and would go untreated [28]. Other studies in nonpregnant and pregnant women in the South African setting have drawn similar conclusions and question the effectiveness of current syndromic management guidelines with respect to current HIV prevention strategies [29, 30]. A recent systematic review and meta-analysis was commissioned by the World Health Organisation to assess the performance of various screening algorithms used in the syndromic management of vaginal and cervical infections [31]. The authors reported that the vaginal discharge chart that included history and risk assessment worked well in nonpregnant women but was not effective in pregnant women with vaginal discharge. With a positive predictive value (PPV) of 50%, 1 in 2 pregnant women presenting with vaginal discharge were more likely to be overtreated because the vaginal discharge was more likely to be as a result of vaginal candidiasis [28, 31]. Our findings of a similar PPV (48%) for vaginal discharge in South African pregnant women are also suggestive of overtreatment. However, we did not test for other reproductive infections so we were unable to identify the cause of the abnormal vaginal discharge.

Another important finding from our study has further relevance to current treatment guidelines for bacterial vaginosis. An estimated 12% of HIV negative pregnant women had severe bacterial vaginosis (Nugent score 9-10) and only 56% presented with abnormal vaginal discharge. These findings if translated to treatment practice would mean that 40-50% of pregnant women with severe BV would go untreated throughout pregnancy. Early and adequate treatment of severe BV could prevent adverse pregnancy outcomes and prevent HIV acquisition during pregnancy. In one of the largest community based STI intervention studies in the Rakai District of Uganda, women with severe BV were twice more likely to be infected with HIV-1 when compared to women with normal vaginal microbiota (OR 2.08; 95%CI 1.48-2.94) [10]. In a nested case control study of HIV infected South African women, BV was diagnosed in more than 70% of women who had recently seroconverted [30]. Authors concluded after adjustment for other risk factors and in comparison to a HIV uninfected control group that BV contributed to a fair number of new HIV infections and with this evidence they call for current syndromic management guidelines for BV to be reexamined.

As a limitation to our study findings the Nugent scoring system quantifies the abundance of the different bacterial morphotypes but does not distinguish between bacterial species such as* Lactobacillus crispatus* versus* Lactobacillus iners*. Vaginal pH testing also has its limitation because reading the calorimetric pH strip is subjective.

In conclusion, the high prevalence of asymptomatic BV in HIV uninfected pregnant women in a high HIV burden setting has important implications for HIV prevention during pregnancy and adverse pregnancy outcomes if BV is not promptly and adequately treated. There is certainly a need for evaluations of inexpensive point-of-care testing methods that are superior in sensitivity and specificity to vaginal pH testing alone for the correct and prompt diagnosis of BV in pregnant women. For now, a combination of vaginal pH testing and the Whiff test should be evaluated in other studies to confirm the superior performance in the prompt diagnosis of BV as reported previously. Furthermore, these studies should also be designed to determine the impact of prompt treatment of BV in pregnancy on HIV acquisition and adverse pregnancy outcomes, hence validating the importance of diagnosing BV in pregnancy.

## Figures and Tables

**Table 1:** Severity of bacterial vaginosis by Nugent score in relation to prevalence, proportion symptomatic, and the mean vaginal pH in HIV uninfected pregnant women.

Variable	Nugent Score	Prevalence N (%; 95%CI)	Symptomatic (Abnormal Vaginal Discharge) N (%)	Vaginal pH Mean±SD pH>4.4 N (%)
Severe Bacterial Vaginosis	9-10	90 (12.0; 9.9-14.5)	51 (56.7)	4.8±0.6 63 (70.0)

Moderate Bacterial Vaginosis	7-8	190 (25.3; 22.4-28.6)	97 (51.1)	4.8±0.6 119 (64.0)

Intermediate	4-6	121 (16.1; 13.7-18.9)	48 (39.7)	4.7±0.6 63 (53.4)

Normal	0-3	349 (46.5; 42.9-50.1 )	104 (29.8)	4.4±0.5 114 (33.4)

**Table 2:** Demographic and behavioural correlates of bacterial vaginosis in HIV negative pregnant women.

Variable	Level	Bacterial Vaginosis (N=280) n (%)	Intermediate (N=121) n (%)	Normal (N=349) n (%)	P-value
Age (Years)	<19	59 (21.1)	22 (18.2)	99 (28.4)	0.037*∗*
	20 – 24	122 (43.6)	50 (41.3)	118 (33.8)	
	>24	99 (35.4)	49 (40.5)	132 (37.8)	

Gravidity	1	147 (52.7)	64 (52.9)	175 (50.3)	0.891
	2 - 3	120 (43.0)	50 (41.3)	158 (45.4)	
	>3	12 (4.3)	7 (5.8)	15 (4.3)	
	Missing*∗*	1(.)	0(.)	1(.)	

Gestational age (weeks)	≤14	97 (34.8)	44 (36.7)	117 (33.7)	0.843
	15 - 28	182 (65.2)	76 (63.3)	230 (66.3)	
	>28*∗∗*	0(0)	1 (.)	2 (.)	

Sexual Partner Age	<19	11 (3.9)	6 (5.0)	20 (5.7)	0.172
	20-24	78 (27.9)	24 (19.8)	106 (30.4)	
	>25	191 (68.2)	91 (75.2)	223(63.9)	

Relationship	Living together	31 (11.0)	18 (14.9)	49 (14.1)	0.506
	Not Living together	235 (83.9)	93 (76.9)	283 (81.3)	
	Do not currently have a partner	14 (5.0)	10 (8.3)	16 (4.6)	
	Missing *∗*	0(.)	0(.)	1 (.)	

Frequency of unprotected sex acts in the last 3 months	Mean ± SD	10.5 ± 11.6	9.9 ±10.5	9.6 ±10.3	0.563

Number of Lifetime Sex Partners	Mean ± SD	1.2 ± 0.7	1.1 ±0.4	1.1 ± 0.4	0.416

Duration of Current Relationship (Yrs)	Mean ± SD	3.4 ± 3.2	4.2 ± 4.1	4.0 ± 3.7	0.123

*∗*Missing data (not included in the percentage calculation).

*∗∗* Women with a gestational age >28 weeks were not included in this analysis.

## Data Availability

The data used to support the findings of this study are available from the corresponding author upon request.
